# Interest and Transformative Experience as Predictors of Geoscience Academic and Career Choice

**DOI:** 10.3390/bs15020233

**Published:** 2025-02-18

**Authors:** Amanda D. Manzanares, Kevin J. Pugh

**Affiliations:** School of Psychological Sciences, University of Northern Colorado, Greeley, CO 80639, USA; kevin.pugh@unco.edu

**Keywords:** transformative experience, science education, interest, academic and career choice, geosciences

## Abstract

Recruitment and retention of students in STEM fields continues to be a challenge. Existing models of recruitment and retention emphasize the role of domain interest and identity. In the current research, we investigated the role of transformative experience combined with domain interest/identity in predicting academic and career choice. Transformative experiences represent a form of deep engagement in which students actively apply school learning in their everyday lives and find value in doing so. We looked specifically at academic and career choice, i.e., available educational paths and various career options, in the field of geoscience, as the geosciences currently struggle to attract and retain majors, resulting in a lack of professionals to fill these jobs. We collected survey data from students (n = 60) at three U.S. universities, and used hierarchical multiple regression to investigate self-efficacy, pre-geoscience interest/identity, transformative experience, and post-geoscience interest/identity as predictors of geoscience academic and career choice. The full regression model explained 69% of the variance in geoscience academic/career choice. Further, stepwise regression analysis revealed that post-geoscience interest/identity fully mediated the relations between the other significant predictors (pre-geoscience interest/identity and transformative experience) and geoscience academic/career choice.

## 1. Introduction

With new growth and changes in business practices, companies and industries aim to hire graduates in a variety of STEM fields (Ainslie & Huffman, 2019; Noonan, 2017), including geoscience (Gramling, 2008; LaDue & Pacheco, 2013). However, there are significant concerns about recruitment and retention in STEM (e.g., Seymour & Hunter, 2019), and, in particular, universities struggle to attract and retain geoscience majors, resulting in fewer geoscience majors, minors, and professionals (Barber et al., 2010; Bernard & Cooperdock, 2018; Huntoon & Lane, 2007; LaDue & Pacheco, 2013; Lewis & Baker, 2010; McConnell & van Der Hoeven Kraft, 2011; van der Hoeven Kraft et al., 2011; Viskupic et al., 2022).

In line with Lent et al. (1994, 2018) social cognitive career theory, researchers have found geoscience interest and identity to be a central factor in predicting geoscience recruitment (e.g., Houlton, 2010; LaDue & Pacheco, 2013), impacting individuals’ pursuit of a degree and/or in pursuit of a career in the geosciences (Callahan et al., 2017; Kortz et al., 2020; McConnell & van Der Hoeven Kraft, 2011; van der Hoeven Kraft, 2017; van der Hoeven Kraft et al., 2011). Recently, Pugh et al. (2021) expanded on this research by showing that transformative experiences helped to explain geoscience students’ academic and career choice, even accounting for initial domain interest. Transformative experiences are experiences in which students use school learning to see and experience the world in meaningful new ways in their everyday, out-of-school lives (Pugh, 2011).

However, questions remain about the relation between domain interest and identity and transformative experience, and their predictions of academic and career choice. Pugh et al. (2021) found pre-existing interest and identity to be related to academic and career choice partially through transformative experience, with transformative experience being a significant direct predictor of academic and career choice. However, the study did not include a post-measure of interest and identity. It is possible that transformative experience is related to academic and career choice, partially or fully, through post-interest and identity. Accordingly, the purpose of the current research was to (a) test the replicability of the Pugh et al. (2021) results in the domain of geoscience across multiple institutions, and (b) expand the investigation of geoscience interest and identity and transformative experience as predictors of academic and career choice by adding a post-measure of interest and identity.

### 1.1. Social Cognitive Career Theory

Drawing on Bandura’s (1986) social cognitive theory, Lent et al. (1994) developed social cognitive career theory (SCCT) to understand students’ academic and career development. Lent et al. (1994, 2018) stressed that students exercise personal agency as they determine their career choices, as shown by such factors as interest, identity, and self-efficacy being important predictors of students’ academic and career choice (e.g., Borget & Gilroy, 1994; Lent et al., 1994; Wang & Degol, 2013). Furthermore, a meta-analytic path analysis of 143 studies in the domain of STEM found domain interest to be the strongest predictor of academic and career choice, with self-efficacy a significant indirect predictor through interest (Lent et al., 2018). Drawing on the SCCT model, Pugh et al. (2021) recently confirmed the importance of domain interest and identity as a predictor of academic and career choice, specifically within the domain of geoscience. They also found a perceived connection to instructors and transformative experiences to be important predictors of academic and career choice.

The current research tested whether these results could be replicated, and further explored the relation between domain interest and identity, self-efficacy, and transformative experience as predictors of academic and career choice within geoscience, by adding a post-measure of domain interest and identity. Below, we conceptualize each of these constructs and review the research on their roles as predictors of academic and career choice, particularly within the geosciences.

### 1.2. Geoscience Interest and Identity

Individual interest refers to an enduring desire to re-engage with a particular topic or academic field (Hidi & Renninger, 2006). When centered on an academic field, it is also referred to as domain interest, and encompasses feeling of liking and enjoyment for the domain, along with value for the importance and meaning of the domain (Hidi & Renninger, 2006). In general, individual interest is posited to develop from situational interest, which is interest that is nurtured by the environment in a particular moment, and requires active involvement of the individual and the belief that the content has meaning (Hidi & Renninger, 2006; Pugh et al., 2021).

Students with individual interest in the geosciences have a lasting interest and enjoyment of the geosciences, and find learning geological concepts useful (Pugh et al., 2021). Researchers have found that students cite interest in science generally and interest in geoscience specifically as dominate reasons for selecting a geoscience major (Hoisch & Bowie, 2010; Holmes & O’Connell, 2003; Houlton, 2010; LaDue & Pacheco, 2013; Levine et al., 2007; Sexton et al., 2018).

Identity refers to one’s sense of self, i.e., “this is who I am”, as well as possible selves, i.e., “this is who I could become” (Markus & Nurius, 1986). As with interest, identity can be focused on an academic field, and has been linked to academic and career choice (Lent et al., 1994, 2018; Wigfield & Eccles, 2000). Identity is intertwined with interest, as one’s sense of self is affirmed by partaking in enjoyable and interesting tasks (Wigfield & Eccles, 2000). Science identity and interest has the propensity to re-spark learners’ curiosity about particular content, either in or out of school (Hidi & Renninger, 2006; Markus & Nurius, 1986). Indeed, Pugh et al. (2021) found that measures of geoscience interest and identity did not separate in factor analysis. They further found that a combined geoscience interest/identity measure administered within the first two weeks of the semester was a strong predictor of intent to major in geoscience, confidence in a geoscience major, and intent to pursue a geoscience career.

In the current study, we investigated whether these results could be replicated. In addition, we added an end-of-semester geoscience interest/identity assessment to more fully evaluate the relation between geoscience interest/identity and geoscience academic and career choice, as well as to further explore the relation between transformative experience and geoscience academic and career choice.

### 1.3. Transformative Experiences

Dewey’s (1902/1990, 1933, 1958, 1934/1980, 1938, 1929/1988) pragmatist philosophy of education proposes a theory of experience, and advocates the enrichment and expansion of everyday experience as a central purpose of education. Drawing on this philosophy, Pugh and colleagues (e.g., Pugh, 2011) developed transformative experience theory within the domain of science education. *Transformative experience* concerns students’ engagement with content extending beyond the classroom, and is defined by three characteristics: (1) motivated use (applying content in everyday life when not required to do so), (2) expansion of perception (viewing the world through the lens of curricular content in everyday life), and (3) experiential value (valuing the content for how it expands perception, and developing a deeper appreciation for things that are “re-seen”) (Pugh, 2011). For example, a middle school student demonstrated motivated use by applying meteorology content in her everyday life. She commented, “I think about weather all the time, like I said, I can’t get it out of my head. I can’t help but think about it” (Pugh et al., 2017, p. 387). She demonstrated expansion of perception by explaining how she perceived clouds and weather events through the lens of the science content, and expressed experiential value by stating that meteorology content is “good information…because it happens in my everyday life, and it affects me and other people. So that’s what’s cool about it” (Pugh et al., 2017, p. 388).

Transformative experiences have been linked to interest and identity. Some studies have found domain interest and/or identity to be predictive of transformative experience (Pugh, 2004; Pugh et al., 2019a), and other studies have found transformative experience to be predictive of interest and positive emotions (e.g., Heddy & Sinatra, 2013; Heddy et al., 2016). Transformative experiences have also been linked to academic and career choice. Pugh et al. (2019b) found that among female students who were non-majors in geoscience departments, those graduating with a higher percentage reported higher levels of transformative experience than those graduating with a lower percentage. In a path analysis, Pugh et al. (2021) found significant direct paths from transformative experience to geoscience academic and career choice, and indirect paths from pre-existing geoscience interest/identity to geoscience academic and career choice, through transformative experience for female but not male students. They suggested that transformative experiences may help to offset discrimination experienced by female students in geoscience, but cautioned that research is needed before drawing conclusions.

**Figure 1:**
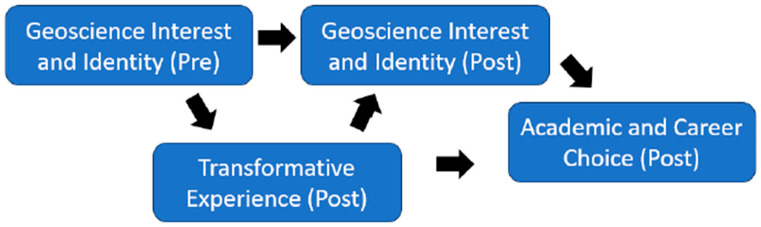
Hypothesized links between constructs.

However, more research is needed in order to understand the relations between transformative experience, geoscience interest/identity, and geoscience academic and career choice. One question that remains is whether transformative experience directly predicts academic and career choice, or whether this relation is mediated by end-of-semester geoscience interest/identity. Accordingly, we included an end-of-semester measure of geoscience interest/identity. Such a measure was not included by Pugh et al. (2021). We did not include a pre-measure of transformative experience, as transformative experience focuses on out-of-school engagement with particular content learned in a school context. Many of the participants in the current study likely had not taken a prior geoscience course in college, and, thus, a pre-measure would not have been valid. Figure 1 illustrates the hypothesized relations between variables, based on the literature reviewed previously.

### 1.4. Self-Efficacy

Self-efficacy is a measurement of students’ performance capabilities, that is, how confident students feel regarding completing particular tasks (Bandura, 1982; Linnenbrink-Garcia & Patall, 2016; Zimmerman, 2000). Zimmerman (2000) found low self-efficacy to be an impediment to students to pursuit of a particular academic and/or career path. However, in the meta-analytic path analysis conducted by (Lent et al., 2018), self-efficacy was found to be a predictor of interest, rather than a direct predictor of academic and career choice. Accordingly, in the current study, we included pre-existing geoscience self-efficacy as a control in our model of predictors of geoscience academic and career choice.

### 1.5. Current Study

The purpose of the current research was to investigate pre-existing (pre) geoscience interest/identity, end-of-semester (post) geoscience interest/identity, and transformative experience as predictors of geoscience academic and career choice, controlling for geoscience self-efficacy, in the context of introductory geoscience courses. We used hierarchical multiple regression for this investigation, resulting in the following research questions and hypotheses.

RQ1: Controlling for self-efficacy, is pre-geoscience interest/identity a significant predictor of geoscience academic/career choice?

H1: Based on prior research (e.g., Lent et al., 2018), we hypothesized that pre-geoscience interest/identity would be a significant and strong predictor of geoscience academic/career choice in step one of the regression analysis.

RQ2: Does transformative experience contribute significant variance in explaining geoscience academic/career choice beyond that accounted for by self-efficacy and pre-geoscience interest/identity?

H2: Based on prior research (e.g., Pugh et al., 2021), we hypothesized that transformative experience would be a significant predictor when added in step two of the regression analysis, and would contribute significant variance in explaining geoscience academic/career choice beyond the step one predictors.

RQ3: Does post-geoscience interest/identity contribute significant variance in explaining geoscience academic/career choice beyond that accounted for by self-efficacy, pre-geoscience interest/identity, and transformative experience?

H3: We are unaware of research predicting academic and career choice with pre- and post-measures of interest/identity. Nevertheless, we predicted that post-geoscience interest/identity would be a significant predictor in step three of the regression analysis, and would contribute additional variance. We made this prediction based on the assumption that geoscience interests and identity beliefs are likely to coalesce and become more defined as students take an introductory geoscience course. Thus, their end-of-semester interest/identity should be a stronger predictor than their pre-existing interest/identity. We also predicted that post-geoscience interest identity would explain variance in academic and career choice beyond that explained by transformative experience; however, this relation has not been investigated in prior research.

RQ4: Are the relations of pre-geoscience interest/identity and transformative experience to geoscience academic/career choice fully or partially mediated through post-geoscience interest/identity?

H4: Based on the strong connection between pre- and post-geoscience interest/identity, we predicted that the relation between pre-geoscience interest/identity and geoscience academic/career choice would be fully mediated by post-geoscience interest/identity. That is, higher levels of pre-geoscience interest/identity were expected to predict higher levels of post-geoscience interest/identity, and this association was predicted to explain the connection between pre-geoscience interest/identity and geoscience academic/career choice. Finally, based on the finding that transformative experience is predictive of interest (e.g., Heddy & Sinatra, 2013), we predicted that the relation between transformative experience and geoscience academic/career choice would be at least partially mediated by post-geoscience interest/identity. That is, higher levels of transformative experience were predicted to be associated with higher levels of post-geoscience interest/identity, and this relation was predicted to at least partially explain the connection between transformative experience and geoscience academic/career choice.

## 2. Materials and Methods

### 2.1. Participants

Participants included undergraduate students enrolled in geoscience courses at three universities in the United States (Table 1). Students were recruited by introductory geoscience course instructors, and received extra credit as compensation, at the discretion of their instructor.

Sixty students completed the pre- and post-survey. Thirty-seven students (62%) identified as female, twenty-two (37%) as male, and one as non-binary. Age ranged from 18 to 38 (M = 19.2). Regarding race/ethnicity, 83% of the students identified as white, 8.3% as Asian, 5% as more than one race, 1.7% as black, and 1.7% as another race. Furthermore, 8.3% of the students identified as Hispanic or Latino/Latina.

A priori power analysis was conducted using G*Power version 3.1.9.7 (Faul et al., 2007) to determine the minimum sample size required to test our research questions. To detect a large effect (f^2^ = 0.35) with four predictors in a hierarchical multiple regression model, at a significance level of α = 0.05 and with 95% power, a sample size of approximately 34 participants is required. Therefore, the obtained sample size of n = 60 meets the sample size for these requirements.

### 2.2. Procedure

An online pre-survey containing measures of geoscience interest/identity and self-efficacy, along with demographic questions, was completed by participants in the first two weeks of the semester. It contained a total of 19 items, and took 10–15 min to complete. An online post-survey containing measures of transformative experience, geoscience interest/identity, and geoscience academic/career choice was completed by participants in the last two weeks of the semester. It contained a total of 32 questions, and took about 20–30 min. Participants were provided with a link to the surveys by their course instructor, and completed them in their own time, outside of class.

### 2.3. Measures

In line with prior use of the measures, the geoscience interest/identity scale and self-efficacy scale used a 5-point Likert scale (1 = strongly disagree, 5 = strongly agree), the transformative experience measure used a 4-point Likert scale (1 = strongly disagree, 4 = strongly agree), and the geoscience academic/career choice scale used a 7-point Likert scale (1 = strongly disagree, 7 = strongly agree). Sample items are provided in Table 2.

All measures were adapted from previously developed and validated measures. As a reliability check, we reported the alpha for each measure, except the Transformative Experience Questionnaire (TEQ). The TEQ was developed and validate using Rasch, and, thus, we used Rasch methods to establish the reliability and validity of this measure. Further, we used exploratory factor analysis (EFA) to investigate whether the geoscience self-efficacy, geoscience interest, and geoscience identity items separated at pre, and whether the geoscience interest and geoscience identity items separated at post (there was no post-geoscience self-efficacy measure). The TEQ items were not included in the EFA, because the measure was designed for use with Rasch analysis. Results from the EFA at pre revealed three factors with an eigenvalue at or above one (geoscience self-efficacy, geoscience interest, geoscience identity), with minimal crossloading between factors. However, on the post EFA, we only found a single factor with an eigenvalue greater than one. When we forced a two-factor solution, one geoscience identity item moderately crossloaded (0.39) on the geoscience interest factor. Given the strong correlation between geoscience interest and geoscience identity at pre (0.71, *p* < 0.001) and post (0.76, *p* < 0.001), we decided to follow previous use of the scale (Pugh et al., 2021) and create a combined geoscience interest/identity scale. When combined, all the geoscience interest and identity items loaded on the combined factor at or greater than 0.68 (pre) and 0.77 (post), and the combined scales had strong reliability, as reported below.

#### 2.3.1. Geoscience Self-Efficacy

The measure of geoscience self-efficacy was adapted from PALS (Midgley et al., 2000). Students’ self-efficacy was measured with four items. The 4-item scale had good reliability (α = 0.77).

#### 2.3.2. Geoscience Interest/Identity

The geoscience interest/identity measure was adapted from Pugh et al. (2021). The same measure was used for the pre- and post-survey. The 9-item scale targeted students’ liking and value for geoscience (i.e., interest; five items), and feeling of connection and belonging in geoscience (i.e., identity; four items). The scale had strong reliability (pre α = 0.93; post α = 0.94).

#### 2.3.3. Transformative Experience Questionnaire

Transformative experience was assessed using an adapted version of the Transformative Experience Questionnaire (Koskey et al., 2018). The 16-item scale assessed the three characteristics of transformative experience: motivated use (seven items), expansion of perception (four items), and experiential value (five items). In addition, the measure contained items targeting a continuum of transformative engagement (Pugh et al., 2010), ranging from in-class engagement (e.g., “During geology class, I notice examples of geoscience”), to out-of-class engagement (e.g., “I notice examples outside of class of geoscience”), to active out-of-class engagement (e.g., “I look for examples outside of class of geoscience”). Per precedent, Rasch (1980) analysis was used to evaluate the measure and develop a composite score. All item infit values were within the 0.60 to 1.40 cutoff recommended by Wright and Linacre (1994). Further, the person reliability (0.93) and separation (3.67) were strong, indicating a reliable ordering of participants along a continuum of low to high transformative engagement, and indicating that the measure distinguished between different groups of participants. In addition, the item reliability (0.92) and separation (3.42) were strong.

#### 2.3.4. Academic/Career Choice

The academic and career choice scale was adapted from (Pugh et al., 2021). Pugh et al. (2021) used separate scales for geoscience majors and non-majors. We combined these and created items applicable to both majors and non-majors. Three items targeted students’ inclination to major or minor in geoscience (i.e., academic choice), and three items targeted their inclination to pursue a career in geoscience (i.e., career choice). The 6-item scale had strong reliability (α = 0.98).

### 2.4. Analysis

Hierarchical multiple regression was used to investigate self-efficacy, pre-geoscience interest/identity, transformative experience, and post-geoscience interest/identity as predictors of geoscience academic and career choice. Further details of the analysis are presented with the results.

## 3. Results

### 3.1. Descriptive Statistics

Table 3 provides the means and standard deviations for the five constructs used in our model. The geoscience interest/identity means were, on average, at the neutral level. Self-efficacy was, on average, at the agree level, meaning that, on average, students agreed to the self-efficacy items. For geoscience academic/career choice, students typically answered with somewhat disagree, or the neutral stance of neither agree nor disagree. The transformative experience mean was high. Rasch analysis orders items from easiest to hardest to endorse, thus creating a continuum from lower to higher levels of transformative engagement. In line with prior results (e.g., Koskey et al., 2018), items targeting in-class engagement were generally easiest to endorse, while items targeting active out-of-class engagement were generally hardest to endorse. Students in our sample were likely to endorse even the hardest items, indicating that students, on average, were experiencing high levels of transformative experience in their geoscience courses.

The correlations, also in Table 3, indicate that the strongest correlation with academic/career choice was post-interest/identity. This was followed by transformative experience and pre-interest/identity. Self-efficacy showed no correlation with the other variables.

### 3.2. Regression Results

Prior to conducting the regression analysis, the predictor variables were centered to reduce multicollinearity. No variables in the regression model exceeded the multicollinearity cutoffs of Tolerance < 0.25 and VIF > 4. Table 4 presents the hierarchical multiple regression results. In step one, self-efficacy and pre-geoscience interest/identity were entered. Together, they accounted for 43% of the variance in geoscience academic/career choice. However, self-efficacy was not a meaningful or statistically significant predictor. In contrast, pre-geoscience interest/identity was a large and statistically significant predictor. In step two, transformative experience was added to the regression model. It accounted for an additional 14% of the variance, and was a statistically significant predictor, with a medium effect size. Thus, controlling for students’ existing domain interest/identity and self-efficacy, transformative experience was still an important predictor of academic and career choice. In step three, post-geoscience interest/identity was added to the regression model. It accounted for an additional 12% of the variance, and was a statistically significant predictor, with a large effect size. The full regression model accounted for an impressive 69% of the variance in geoscience academic/career choice.

In step three, pre-geoscience interest/identity and transformative experience were no longer statistically significant predictors. Given that both variables were (a) significant predictors before post-geoscience interest/identity was added and (b) significantly correlated with post-geoscience interest/identity, we can assume full mediation, according to criteria established by Baron and Kenny (1986). That is, students with higher levels of pre-existing geoscience interest/identity and students undergoing higher levels of transformative experience were more likely to have higher levels of geoscience interest/identity near the end of the semester. This increased geoscience interest/identity at post-test explains the positive relation of pre-geoscience interest/identity and transformative experience to academic/career choice.

## 4. Discussion

In line with our hypothesis and prior research (e.g., LaDue & Pacheco, 2013; Lent et al., 2018; Pugh et al., 2021; Sexton et al., 2018), pre-geoscience interest/identity was a strong predictor of geoscience academic/career choice, controlling for self-efficacy. Also, in line with our hypothesis and prior research (Pugh et al., 2021), in step two of the regression analysis, transformative experience contributed to explaining variance in geoscience academic/career choice beyond that explained by pre-geoscience interest/identity and self-efficacy. Thus, accounting for students’ pre-existing sense of efficacy in the domain of geoscience, and their pre-existing interest in and identification with the domain, students who more strongly endorsed experiences of applying geoscience in their everyday lives and using it to see the world in meaningful new ways were more likely to report a higher inclination to pursue a major and career in geoscience. In addition, in step three of the regression analysis, post-geoscience interest/identity contributed to explaining variance in geoscience academic/career choice beyond that explained by transformative experience, pre-geoscience interest/identity, and self-efficacy. This result was also in line with our hypothesizing.

To further understand these results, we investigated the possibility of mediation. As explained in the results, we found evidence for mediation according to criteria established by Baron and Kenny (1986). Specifically, as predicted, the relation between pre-geoscience interest/identity and geoscience academic/career choice was fully mediated by post-geoscience interest/identity. This result is not surprising, and it simply confirms the critical role of domain interest/identity in predicting academic and career choice, as has been established in prior research (e.g., Lent et al., 2018), including research in the domain of geoscience (e.g., LaDue & Pacheco, 2013; Pugh et al., 2021).

Of greater interest are the mediation results related to transformative experience. We predicted at least partial mediation, and found that the relation between transformative experience and geoscience academic/career choice was fully mediated by post-geoscience interest/identity. This result contributes to our understanding of how transformative experience relates to academic and career choice. Pugh et al. (2021) only included a pre-measure of interest/identity, and found transformative experience to be a significant direct predictor of academic and career choice. The current study included a post-measure of interest/identity, and we found evidence of a mediational relation, instead of a direct one. That is, controlling for pre-existing domain interest/identity, transformative experience is positively related to end-of-semester domain interest/identity, and end-of-semester interest/identity is predictive of higher levels of academic and career choice.

### 4.1. Limitations and Future Directions

The current study relied on correlational data. Future experimental research is needed to establish causality. Furthermore, our research relied on self-report, and students may not be able to reflect on their motivational and experiential states with full accuracy. Generalizability limitations stem from the sample. A majority of participants identified as female, which likely reflects introductory geoscience course demographics, but differs from male-majority geoscience major and career demographics in the U.S. (National Science Foundation, National Center for Science and Engineering Statistics, 2015, 2017). Additionally, a lesser proportion of participants completed both the pre- and post-survey and were included in the study. It is possible these participants differ from those that would be found in larger samples (e.g., more diligent students or more concerned about extra credit). Future research with a larger and more diverse sample is also needed to explore whether the results found in the current study are maintained across gender, race/ethnicity, and other demographic categories. Given that women and minority populations are under-represented in STEM fields, and particularly in geoscience (National Science Foundation, National Center for Science and Engineering Statistics, 2015), it is important for future research to investigate predictors of academic and career choice specifically for these populations. Future research could also include a pre-measure of academic and career choice to control for students’ pre-existing inclinations to pursue a major or career in geoscience.

### 4.2. Implications

The current research confirmed the strong relation that interest/identity and transformative experience have to academic and career choice in geoscience. Although the results of the current study are not causal, they suggest that instructors may be able to address concerns with recruitment and retention in geoscience (Huntoon & Lane, 2007; Wolfe, 2018) by fostering transformative experiences and domain interest and identity in introductory geoscience courses. A full review of how to foster these outcomes is beyond the scope of the current research. Nevertheless, we highlight a few key sources. In a review of the research, Bergin (1999) identified influences on interest, including, but not limited to, factors such as novelty, discrepancy, narrative, and sense of belonging. Others (Hidi & Renninger, 2006; Renninger & Hidi, 2017) have proposed a four-phase model of interest development in which situational interests (i.e., interests developed and supported in the classroom) lead to enduring individual and domain interest. Factors that foster situational interest include involvement, meaning, and relevance. Domain identity can be supported by providing students with access to mentors and role models (Packard & Nguyen, 2003) and engaging students in enjoyable and interesting tasks (Lent et al., 1994, 2018). Finally, based on research focused on fostering transformative experiences, Pugh (2020) developed the Teaching for Transforming Experiences in Science (TTES) model. This model includes strategies such as artistically crafting the content, scaffolding students’ re-seeing of the world through the lens of curricular content, and modeling one’s own passion for the content.

## 5. Conclusions

Recruitment and retention of individuals in STEM fields is a global concern (van Tuijl & van der Molen, 2016), and the geosciences have particularly struggled to recruit and retain students (Barber et al., 2010; McConnell & van Der Hoeven Kraft, 2011; van der Hoeven Kraft et al., 2011; Viskupic et al., 2022). Drawing on the social cognitive career theory (SCCT) framework (e.g., Lent et al., 1994, 2018), we investigated transformative experience and geoscience interest and identity as predictors of geoscience academic and career choice. Overall, our results suggest that students with a higher level of pre-existing geoscience interest and identity, and students who undergo higher levels of transformative experience in introductory geoscience courses, are more likely to have higher levels of geoscience interest and identity by the end of the semester. Heightened interest in and identification with geoscience at the end of the semester is a strong predictor of students’ inclination to pursue a major and career in geoscience.

## Figures and Tables

**Table 1:** University information and completed surveys per institution.

Institution	Size ^1^	Location ^2^	Pre-Survey (n)	Post-Survey (n)	Both (n)
University 1	~3000	Midwest	33	39	26
University 2	~40,000	Midwest	64	35	24
University 3	~9000	West	13	19	10
Total			110	93	60

Note: ^1^ Undergraduate population. ^2^ Region of United States.

**Table 2:** Survey sample items.

Measure	Sample Item
Pre- and Post-II ^1^	
Interest	I am fascinated by the geosciences.
Identity	I identify with the field of study known as the geosciences.
TEQ ^2^	
Motivated Use	Outside of school, I ***use*** the knowledge I’ve learned about geoscience.
Expansion of Perception	I notice examples outside of class of geoscience.
Experiential Value	I find that knowledge of geoscience makes my current, out-of-school experience more meaningful.
SE ^3^	Even if the work in my geoscience course(s) is hard, I can learn it.
ACC ^4^	
Academic Choice	I can see myself pursuing a geoscience major or minor.
Career Choice	I can imagine myself being involved in a geoscience career.

Note: ^1^ Pre- and post-interest/identity scale. ^2^ Transformative Experience Questionnaire. ^3^ Self-efficacy scale. ^4^ Academic/career choice scale.

**Table 3:** Means, standard deviations, and correlations.

	N	Mean	SD	1	2	3	4
1. Self-efficacy ^1^	60	4.07 ^3^	0.51	--			
2. Pre-geoscience interest/identity ^1^	60	3.27 ^3^	0.75	0.192	--		
3. Transformative experience ^2^	60	2.86 ^4^	0.50	0.025	0.480 ***	--	
4. Post-geoscience interest/identity ^2^	60	3.33 ^4^	0.81	−0.011	0.665 ***	0.815 ***	--
5. Academic/career choice ^2^	60	3.73 ^5^	1.99	0.016	0.644 ***	0.654 ***	0.817 ***

Note: * *p* < 0.05, ** *p* < 0.01, *** *p* < 0.001. ^1^ Pre-survey. ^2^ Post-survey. ^3^ Responses were on a 5-point Likert scale (1 = strongly disagree; 5 = strongly agree). ^4^ Measured in logits; <1.5 = not at all transformative, >1.5 = highly transformative. ^5^ Responses were on a 7-point Likert scale (1 = strongly disagree; 5 = strongly agree).

**Table 4:** Predicting geoscience academic and career choice.

Predictor	B	*SE* B	*β*	*R* ^2^	∆*R*^2^
**Step 1**				0.43	0.43 ***
Self-Efficacy	−0.44	0.40	−0.11		
Pre-Geoscience Interest/Identity	1.75	0.27	0.67 ***		
**Step 2**				0.57	0.14 ***
Self-Efficacy	−0.29	0.32	−0.08		
Pre-Geoscience Interest/Identity	1.23	0.27	0.46 ***		
Transformative Experience	0.39	0.09	0.43 ***		
**Step 3**				0.69	0.12 ***
Self-Efficacy	−0.05	0.31	−0.01		
Pre-Geoscience Interest/Identity	0.50	0.28	0.19		
Transformative Experience	0.01	0.01	0.01		
Post-Geoscience Interest/Identity	1.67	0.37	0.68 ***		

Note: N = 60. * *p* < 0.05. ** *p* < 0.01. *** *p* < 0.001. *β* 0.01–0.29 = small, 0.30–0.49 = medium, 0.50 or greater = large (Cohen, 1988).

## Data Availability

The data that support the findings of this study are available on request from the corresponding author. The data are not publicly available, due to them containing information that could compromise the privacy of research participants.

## References

[B1-behavsci-15-00233] Ainslie P. J., Huffman S. L. (2019). Human resource development and expanding STEM career learning opportunities: Exploration, internships, and externships. Advances in Developing Human Resources.

[B2-behavsci-15-00233] Bandura A. (1982). Self-efficacy mechanism in human agency. American Psychologist.

[B3-behavsci-15-00233] Bandura A. (1986). Social foundations of thought and action: A social cognitive theory.

[B4-behavsci-15-00233] Barber L. D., Pifer M. J., Colbeck C., Furman T. (2010). Increasing diversity in the geosciences: Recruitment programs and student self-efficacy. Journal of Geoscience Education.

[B5-behavsci-15-00233] Baron R. M., Kenny D. A. (1986). The moderator-mediator variable distinction in social and psychological research: Conceptual, strategic, and statistical considerations. Journal of Personality and Social Psychology.

[B6-behavsci-15-00233] Bergin D. A. (1999). Influences on Classroom Interest. Educational Psychologist.

[B7-behavsci-15-00233] Bernard R. E., Cooperdock E. H. (2018). No progress on diversity in 40 years. Nature Geoscience.

[B8-behavsci-15-00233] Borget M. M., Gilroy F. D. (1994). Interests and self-efficacy as predictors of mathematics/science-based career choice. Psychological Reports.

[B9-behavsci-15-00233] Callahan C. N., LaDue N. D., Baber L. D., Sexton J., van der Hoeven Kraft K. J., Zamani-Gallaher E. M. (2017). Theoretical perspectives on increasing recruitment and retention of underrepresented students in the geosciences. Journal of Geoscience Education.

[B10-behavsci-15-00233] Cohen J. (1988). Statistical power analysis for the behavioral sciences.

[B11-behavsci-15-00233] Dewey J. (1933). How we think: A restatement of the relation of reflective thinking to the educative process.

[B12-behavsci-15-00233] Dewey J. (1938). Experience and education.

[B13-behavsci-15-00233] Dewey J. (1958). Experience and nature.

[B14-behavsci-15-00233] Dewey J. (1980). Art as experience.

[B15-behavsci-15-00233] Dewey J., Boydston J. A. (1988). The quest for certainty. John Dewey: The later works, 1925–1953.

[B16-behavsci-15-00233] Dewey J. (1990). The school and society and the child and the curriculum.

[B17-behavsci-15-00233] Faul F., Erdfelder E., Lang A.-G., Buchner A. (2007). G*Power 3: A flexible statistical power analysis program for the social, behavioral, and biomedical sciences. Behavior Research Methods.

[B18-behavsci-15-00233] Gramling C. (2008). In the geosciences, business is booming. Science.

[B19-behavsci-15-00233] Heddy B. C., Sinatra G. M. (2013). Transforming misconceptions: Using transformative experience to promote positive affect and conceptual change in students learning about biological evolution. Science Education.

[B20-behavsci-15-00233] Heddy B. C., Sinatra G. M., Seli H., Taasoobshirazi G., Mukhopadhyay A. (2016). Making learning meaningful: Facilitating interest development and transfer in at-risk college students. Educational Psychology.

[B21-behavsci-15-00233] Hidi S., Renninger K. A. (2006). The four-phase model of interest development. Educational Psychologist.

[B22-behavsci-15-00233] Hoisch T. D., Bowie J. I. (2010). Assessing factors that influence the recruitment of majors from introductory geology classes at Northern Arizona University. Journal of Geoscience Education.

[B23-behavsci-15-00233] Holmes M. A., O’Connell S. (2003). Where are the women geoscience professors?.

[B24-behavsci-15-00233] Houlton H. R. (2010). Academic provenance: Investigation of pathways that lead students into the geosciences. Unpublished Master’s thesis.

[B25-behavsci-15-00233] Huntoon J. E., Lane M. J. (2007). Diversity in the geosciences and successful strategies for increasing diversity. Journal of Geoscience Education.

[B26-behavsci-15-00233] Kortz K. M., Cardace D., Savage B. (2020). Affective factors during field research that influence intention to persist in the geosciences. Journal of Geoscience Education.

[B27-behavsci-15-00233] Koskey K., Stewart V., Sondergeld T., Pugh K. J. (2018). Applying the mixed methods instrument development and construct validity process: The case of the transformative experience questionnaire. Journal of Mixed Methods Research.

[B28-behavsci-15-00233] LaDue N. D., Pacheco H. A. (2013). Critical experiences for field geologists: Emergent themes in interest development. Journal of Geoscience Education.

[B29-behavsci-15-00233] Lent R. W., Brown S. D., Hackett G. (1994). Toward a unifying social cognitive theory of career and academic interest, choice, and performance. Journal of Vocational Behavior.

[B30-behavsci-15-00233] Lent R. W., Sheu H.-B., Miller M. J., Cusick M. E., Penn L. T., Truong N. N. (2018). Predictors of science, technology, engineering, and mathematics choice options: A meta-analytic path analysis of the social–cognitive choice model by gender and Race/ethnicity. Journal of Counseling Psychology.

[B31-behavsci-15-00233] Levine R., González R., Cole S., Fuhrman M., Le Floch K. C. (2007). The Geoscience Pipeline: A Conceptual Framework. Journal of Geoscience Education.

[B32-behavsci-15-00233] Lewis E. B., Baker D. R. (2010). A call for a new geoscience education research agenda. Journal of Research in Science Teaching: The Official Journal of the National Association for Research in Science Teaching.

[B33-behavsci-15-00233] Linnenbrink-Garcia L., Patall E. A., Corno L., Anderman E. M. (2016). Motivation. Handbook of educational psychology.

[B34-behavsci-15-00233] Markus H., Nurius P. (1986). Possible selves. American Psychologist.

[B35-behavsci-15-00233] McConnell D. A., van Der Hoeven Kraft K. J. (2011). Affective domain and student learning in the Geosciences. Journal of Geoscience Education.

[B36-behavsci-15-00233] Midgley C., Maehr M. L., Hruda L. Z., Anderman E., Anderman L., Freeman K. E., Gheen M., Kaplan A., Kumar R., Middleton M. J., Nelson J., Roeser R., Urdan T. (2000). Manual for the patterns of adaptive learning scales (PALS).

[B37-behavsci-15-00233] National Science Foundation, National Center for Science and Engineering Statistics (2015). Earth and ocean science degrees awarded, by degree level and sex of recipient: 1966–2012.

[B38-behavsci-15-00233] National Science Foundation, National Center for Science and Engineering Statistics (2017). Women, minorities, and persons with disabilities in science and engineering: 2017. Special report NSF 17-310.

[B39-behavsci-15-00233] Noonan R. (2017). STEM jobs: 2017 update. ESA issue brief# 02-17.

[B40-behavsci-15-00233] Packard B. W.-L., Nguyen D. (2003). Science career-related possible selves of adolescent girls: A longitudinal study. Journal of Career Development.

[B41-behavsci-15-00233] Pugh K. J. (2004). Newton’s laws beyond the classroom walls. Science Education.

[B42-behavsci-15-00233] Pugh K. J. (2011). Transformative experience: An integrative construct in the spirit of Deweyan pragmatism. Educational Psychologist.

[B43-behavsci-15-00233] Pugh K. J. (2020). Transformative science education: Change how your students experience the world.

[B44-behavsci-15-00233] Pugh K. J., Bergstrom C. M., Heddy B. C., Krob K. E. (2017). Supporting deep engagement: The teaching for transformative experiences in science (TTES) model. The Journal of Experimental Education.

[B45-behavsci-15-00233] Pugh K. J., Bergstrom C. M., Wilson L., Geiger S., Goldman J., Heddy B. C., Cropp S., Kriescher D., Spector J. M., Lockee B. B., Childress M. D. (2019a). Transformative experience: A critical review and investigation of individual factors. Learning, design, and technology: An International compendium of theory, research, practice, and policy.

[B46-behavsci-15-00233] Pugh K. J., Linnenbrink-Garcia L., Koskey K. L. K., Stewart V. C., Manzey C. (2010). Motivation, learning, and transformative experience: A study of deep engagement in science. Science Education.

[B47-behavsci-15-00233] Pugh K. J., Paek S. H., Phillips M. M., Sexton J. M., Bergstrom C. M., Flores S. D., Riggs E. M. (2021). Predicting academic and career choice: The role of transformative experience, connection to instructor, and gender accounting for interest and identity and contextual factors. Journal of Research in Science Teaching.

[B48-behavsci-15-00233] Pugh K. J., Phillips M. M., Sexton J. M., Bergstrom C. M., Riggs E. M. (2019b). A quantitative investigation of geoscience departmental factors associated with the recruitment and retention of female students. Journal of Geoscience Education.

[B49-behavsci-15-00233] Rasch G. (1980). Probabilistic models for some intelligence and attainment tests *(Expanded Ed.)*.

[B50-behavsci-15-00233] Renninger K. A., Hidi S. (2017). The power of interest for motivation and engagement.

[B51-behavsci-15-00233] Sexton J. M., Pugh K. J., Bergstrom C. M., Riggs E. M. (2018). Reasons undergraduate students majored in geology across six universities: The importance of gender and department. Journal of Geoscience Education.

[B52-behavsci-15-00233] Seymour E., Hunter A.-B. (2019). Talking about leaving revisited: Persistence, relocation, and loss in undergraduate STEM education.

[B53-behavsci-15-00233] van der Hoeven Kraft K. J. (2017). Developing student interest: An overview of the research and implications for geoscience education research and teaching practice. Journal of Geoscience Education.

[B54-behavsci-15-00233] van der Hoeven Kraft K. J., Srogi L., Husman J., Semken S., Fuhrman M. (2011). Engaging students to learn through the affective domain: A new framework for teaching in the Geosciences. Journal of Geoscience Education.

[B55-behavsci-15-00233] van Tuijl C., van der Molen J. H. W. (2016). Study choice and career development in STEM fields: An overview and integration of the research. International Journal of Technology and Design Education.

[B56-behavsci-15-00233] Viskupic K., Wenner J. A., Harrigan C. O., Shafer G. (2022). A mixed methods study of the challenges for geoscience majors in identifying potential careers and the benefits of a career awareness and planning course. Journal of Geoscience Education.

[B57-behavsci-15-00233] Wang M. T., Degol J. (2013). Motivational pathways to STEM career choices: Using expectancy–value perspective to understand individual and gender differences in STEM fields. Developmental Review.

[B58-behavsci-15-00233] Wigfield A., Eccles J. S. (2000). Expectancy–value theory of achievement motivation. Contemporary Educational Psychology.

[B59-behavsci-15-00233] Wolfe B. A. (2018). Introductory geosciences at the two-year college: Factors that influence student transfer intent with geoscience degree aspirations. Journal of Geoscience Education.

[B60-behavsci-15-00233] Wright B. D., Linacre J. M. (1994). The rasch model as a foundation for the lexile framework *[Unpublished manuscript]*.

[B61-behavsci-15-00233] Zimmerman B. J. (2000). Self-efficacy: An essential motive to learn. Contemporary Educational Psychology.

